# Supplementary material for: Protection of COVID-19 vaccination and previous infection against Omicron BA.1, BA.2 and Delta SARS-CoV-2 infections
Source: Nat Commun. 2022 Aug 12;13:4738. doi: 10.1038/s41467-022-31838-8 (PMC9373894; doi:10.1038/s41467-022-31838-8)
Supplement: Supplementary file 1 — Supplementary Information [file 41467_2022_31838_MOESM1_ESM.docx]

**Supplementary material**


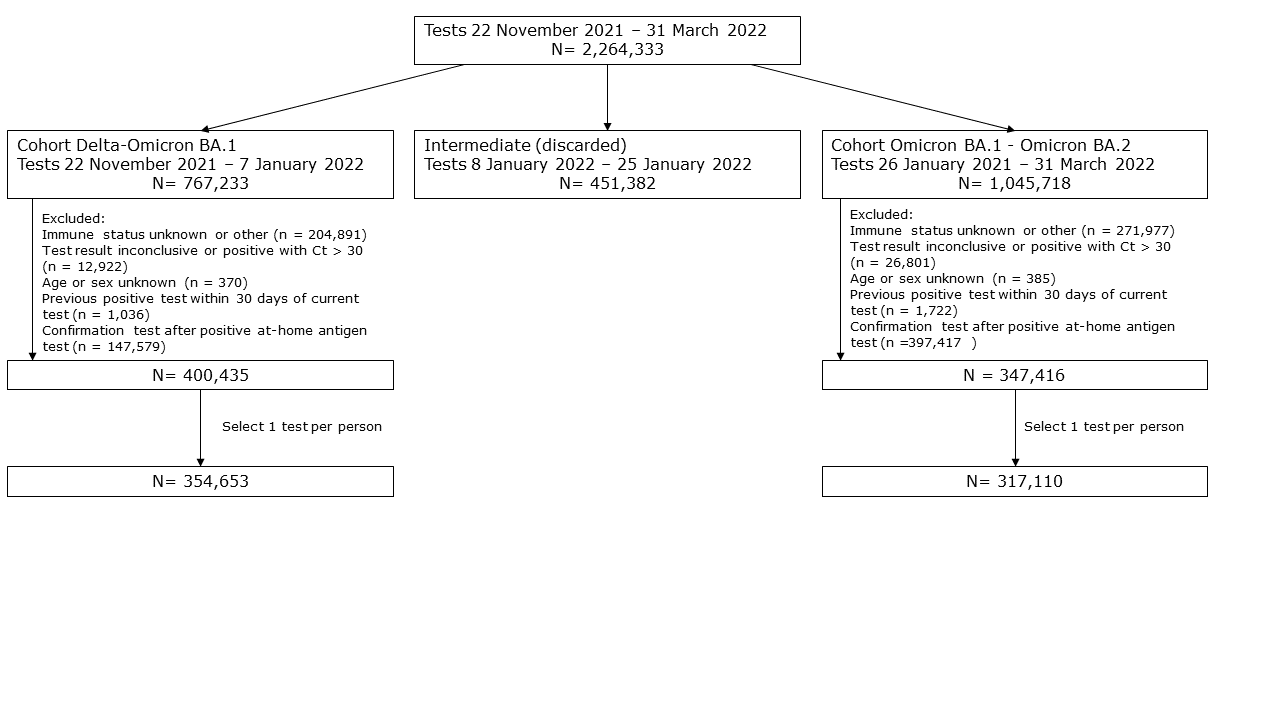


Figure S1. Flowchart of data selection


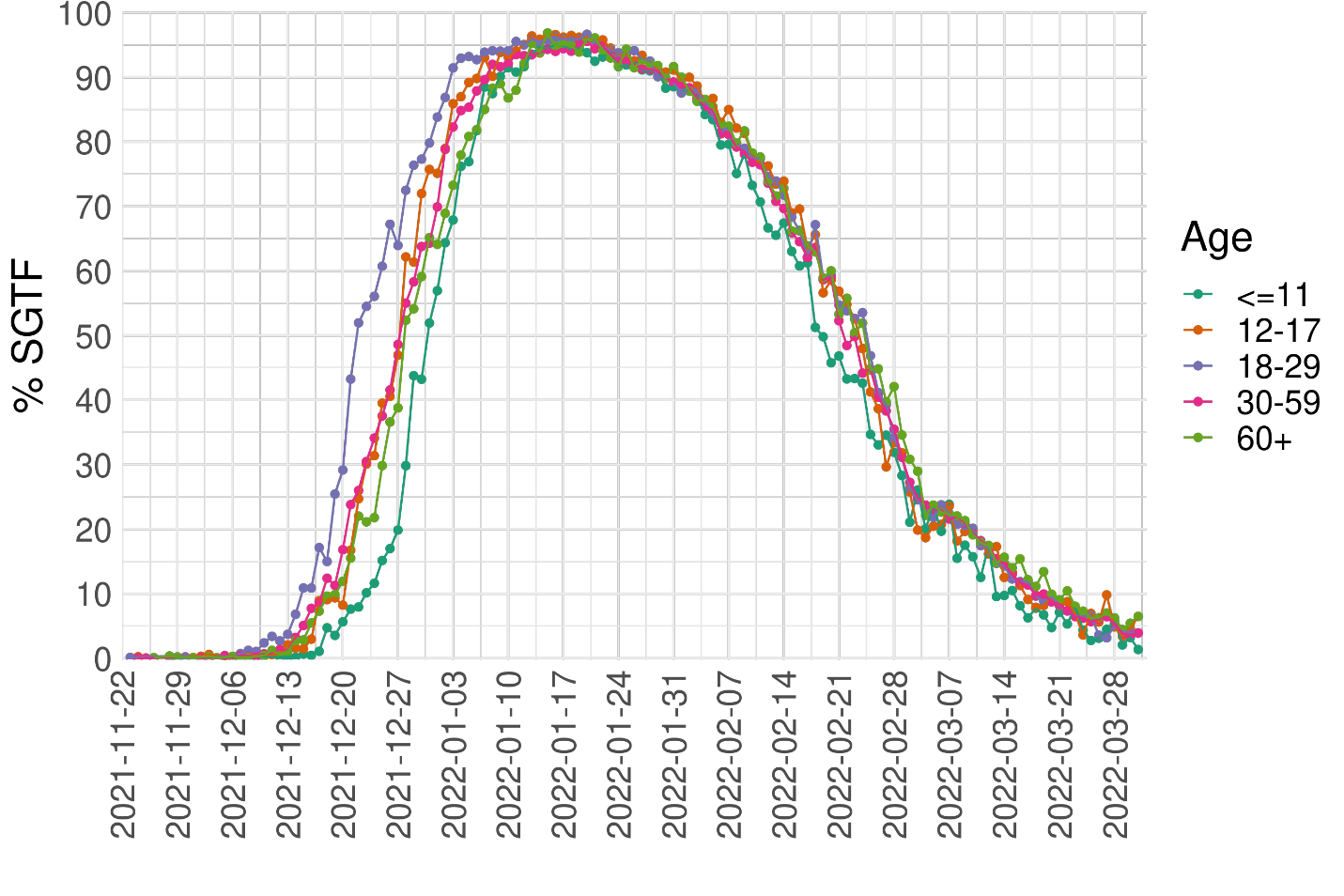


Figure S2. Proportion of SGTF among positive SARS-CoV-2 cases by age group, the Netherlands, 22 November 2021 - 31 March 2022, n = 941,692.

Table S1 WGS variant found in SGTF (S result, not detected) and non-SGTF (S result, detected) cases in each cohort. First column indicates the cohort, followed by the SGTF result. The number of WGS typed Omicron BA.1, Delta and BA.2 cases. Row-wise calculations on positive predictive value (PPV) and column-wise calculations on sensitivity.

| **Cohort** | **S result** | **WGS result**  **Omicron BA.1** | **WGS result**  **Delta** | **WGS result**  **Omicron BA.2** | **PPV** | **Sensitivity** |
| --- | --- | --- | --- | --- | --- | --- |
| Cohort Delta-Omicron BA.1 | Not detected | 132 | 0 | 0 | 1.00 | 1.00 |
| Cohort Delta-Omicron BA.1 | Detected | 0 | 353 | 0 | 1.00 | 1.00 |
|  |  |  |  |  |  |  |
| Cohort Omicron BA.1-BA.2 | Not detected | 158 | 0 | 0 | 1.00 | 0.99 |
| Cohort Omicron BA.1-BA.2 | Detected | 2 | 0 | 128 | 0.98 | 1.00 |

Table S2 Protection estimates for booster vaccination and booster vaccination with a previous infection with 'Primary vaccinated' as reference.

| **Cohort** | **Immuunstatus** | **Variant** | **VE_CI** |
| --- | --- | --- | --- |
| Delta-Omicron BA.1 | Booster | Omicron BA.1 | 46% (47-49) |
| Delta-Omicron BA.1 | Booster | Delta | 75% (72-79) |
| Delta-Omicron BA.1 | Previous infection,  booster | Omicron BA.1 | 58% (46-68) |
| Delta-Omicron BA.1 | Previous infection,  booster | Delta | 96% (85-99) |
| Omicron BA.1-BA.2 | Booster | Omicron BA.1 | 48% (47-49) |
| Omicron BA.1-BA.2 | Booster | Omicron BA.2 | 40% (38-43) |
| Omicron BA.1-BA.2 | Previous infection,  booster | Omicron BA.1 | 70% (68-72) |
| Omicron BA.1-BA.2 | Previous infection,  booster | Omicron BA.2 | 71% (68-73) |
