# Supplementary material for: Protection of COVID-19 vaccination and previous infection against Omicron BA.1, BA.2 and Delta SARS-CoV-2 infections
Source: Nat Commun. 2022 Aug 12;13:4738. doi: 10.1038/s41467-022-31838-8 (PMC9373894; doi:10.1038/s41467-022-31838-8)
Supplement: Supplementary file 3 — Description of Additional Supplementary Files [file 41467_2022_31838_MOESM3_ESM.pdf]

Description of Additional Supplementary Files

File Name: Supplementary Data 1
Description: The relative reduction data underlying Figure 2.

File Name: Supplementary Data 2
Description: The relative reduction data underlying Figure 3.

File Name: Supplementary Data 3
Description: Estimates of interaction between vaccination and previous infection status, and age.

File Name: Supplementary Data 4
Description: Data underlying Figure 1.

File Name: Supplementary Data 5
Description: Accession IDs available in the GISAID database of the WGS data used in this study.
