# Supplementary material for: Protection of COVID-19 vaccination and previous infection against Omicron BA.1, BA.2 and Delta SARS-CoV-2 infections
Source: Nat Commun. 2022 Aug 12;13:4738. doi: 10.1038/s41467-022-31838-8 (PMC9373894; doi:10.1038/s41467-022-31838-8)
Supplement: Supplementary file 8 — Reporting Summary [file 41467_2022_31838_MOESM8_ESM.pdf]

nature portfolio

Corresponding author(s): Mirjam Knol

Last updated by author(s): Jul 1, 2022

Reporting Summary

Nature Portfolio wishes to improve the reproducibility of the work that we publish. This form provides structure for consistency and transparency in reporting. For further information on Nature Portfolio policies, see our Editorial Policies and the Editorial Policy Checklist.

Statistics

For all statistical analyses, confirm that the following items are present in the figure legend, table legend, main text, or Methods section.

n/a	Confirmed	
☐	☒	The exact sample size (n) for each experimental group/condition, given as a discrete number and unit of measurement
☐	☒	A statement on whether measurements were taken from distinct samples or whether the same sample was measured repeatedly
☒	☐	The statistical test(s) used AND whether they are one- or two-sided<br>*Only common tests should be described solely by name; describe more complex techniques in the Methods section.*
☐	☒	A description of all covariates tested
☐	☒	A description of any assumptions or corrections, such as tests of normality and adjustment for multiple comparisons
☐	☒	A full description of the statistical parameters including central tendency (e.g. means) or other basic estimates (e.g. regression coefficient) AND variation (e.g. standard deviation) or associated estimates of uncertainty (e.g. confidence intervals)
☐	☒	For null hypothesis testing, the test statistic (e.g. F, t, r) with confidence intervals, effect sizes, degrees of freedom and P value noted<br>*Give P values as exact values whenever suitable.*
☒	☐	For Bayesian analysis, information on the choice of priors and Markov chain Monte Carlo settings
☒	☐	For hierarchical and complex designs, identification of the appropriate level for tests and full reporting of outcomes
☐	☒	Estimates of effect sizes (e.g. Cohen's d, Pearson's r), indicating how they were calculated

Our web collection on statistics for biologists contains articles on many of the points above.

Software and code

Policy information about availability of computer code

Data collection	Data was extracted from the national community testing register (CoronIT) and linked using an unique sample number (using R version 4.1.2) to SGTF results from two laboratories.
Data analysis	R (version 4.1.2) was used to analyse the data. For data preparation the tidyverse (version 1.3.1) R package collection was used. For statistical analysis the VGAM (version 1.1-5) and splines (version 4.1.2) packages were used.

For manuscripts utilizing custom algorithms or software that are central to the research but not yet described in published literature, software must be made available to editors and reviewers. We strongly encourage code deposition in a community repository (e.g. GitHub). See the Nature Portfolio guidelines for submitting code & software for further information.

Data

Policy information about availability of data

All manuscripts must include a data availability statement. This statement should provide the following information, where applicable:

- Accession codes, unique identifiers, or web links for publicly available datasets
- A description of any restrictions on data availability
- For clinical datasets or third party data, please ensure that the statement adheres to our policy

The data underlying Figure 1 are deposited in Data S4. The relative reduction data underlying Figures 2 and 3 generated in this study have been deposited in the Data S1 and S2 files. The raw case-based data are protected and are not available due to data privacy laws. We could provide aggregated data but not with the level of detail as used in the analysis because of potential for identifiability of individuals. The WGS data used in this study are available in the GISAID database under accession IDs found in the GISAID Acknowledgment Table (DOI: https://doi.org/10.55876/gis8.220701en, GISAID Identifier: EPI_SET_20220701en).

Field-specific reporting

Please select the one below that is the best fit for your research. If you are not sure, read the appropriate sections before making your selection.

☒ Life sciences ☐ Behavioural & social sciences ☐ Ecological, evolutionary & environmental sciences

For a reference copy of the document with all sections, see nature.com/documents/nr-reporting-summary-flat.pdf

Life sciences study design

All studies must disclose on these points even when the disclosure is negative.

Sample size	No statistical method was performed to calculate sample sizes. All tests sampled between 22 November 2021 and 31 March 2022 from two large laboratories using the TaqPath PCR-kit in the Netherlands were used. The start and the end of the study period was dependent on variant circulation (start was 0% Omicron BA.1 infections and the end almost 100% Omicron BA.2). Based on a PPV of >=85% of SGTF for Delta, Omicron BA.1 and BA.2 two cohorts were defined.
Data exclusions	Tests taken in the time period between the two cohorts (8 January - 25 January 2022) were excluded (n = 451,382), because Delta and Omicron BA.2 are indistinguishable using SGTF in this period.<br>For cohort Delta-Omicron BA.1, the following exclusions were made:<br>Immune/vaccination status unknown (n = 204,891), test result inconclusive or positive with Ct < 30 (n = 12,922), age or sex unknown (n = 370), previous positive test within 30 days of current test (n = 1,036), and confirmation test after positive at-home antigen test (n = 147,579). One test per individual was selected (n = 45,782).<br>For cohort Omicron BA.1-BA.2, the following exclusions were made:<br>Immune/vaccination status unknown (n = 271,977), test result inconclusive or positive with Ct < 30 (n = 26,801), age or sex unknown (n = 385), previous positive test within 30 days of current test (n = 1,722), and confirmation test after positive at-home antigen test (n = 397,417). One test per individual was selected (n = 30,306).<br>Flowchart of data selection is displayed in Figure S1.
Replication	Not applicable for this observational study.
Randomization	Not applicable for this observational study.
Blinding	Not applicable for this observational study.

Reporting for specific materials, systems and methods

We require information from authors about some types of materials, experimental systems and methods used in many studies. Here, indicate whether each material, system or method listed is relevant to your study. If you are not sure if a list item applies to your research, read the appropriate section before selecting a response.

Materials & experimental systems

n/a	Involved in the study	
☒	☐	Antibodies
☒	☐	Eukaryotic cell lines
☒	☐	Palaeontology and archaeology
☒	☐	Animals and other organisms
☐	☒	Human research participants
☒	☐	Clinical data
☒	☐	Dual use research of concern

Methods

n/a	Involved in the study	
☒	☐	ChIP-seq
☒	☐	Flow cytometry
☒	☐	MRI-based neuroimaging

Human research participants

Policy information about studies involving human research participants

Population characteristics	The population characteristics are described in Table 1.
Recruitment	Free of charge national community testing is available for Dutch citizens experiencing COVID-19 like symptoms or after contact with someone testing positive for SARS-CoV-2. Test seeking behavior can differ between the groups used in the study. To minimize this bias we exclude individuals who are aware of their SARS-CoV-2 positive status by an at-home rapid antigen test.
Ethics oversight	The Centre for Clinical Expertise at the National Institute for Public Health and the Environment (RIVM) assessed the research proposal following the specific conditions as stated in the law for medical research involving human subjects. The work described was exempted for further approval by the ethical research committee. Pathogen surveillance is a legal task of the

RIVM and is carried out under the responsibility of the Dutch Minister of Health, Welfare and Sports. The Public Health Act (Wet Publieke Gezondheid) provides that RIVM may receive pseudonymised data for this task without informed consent.

Note that full information on the approval of the study protocol must also be provided in the manuscript.
